# Severe Corticosteroid-Induced Ocular Hypertension Requiring Bilateral Trabeculectomies in a Patient with Takayasu's Arteritis

**DOI:** 10.1155/2016/5253029

**Published:** 2016-11-10

**Authors:** Anna Maria Gruener, Pranev Sharma, Sally Ameen, Faisal Ahmed

**Affiliations:** Western Eye Hospital, Imperial College Healthcare NHS Trust, London, UK

## Abstract

We present a rare case of severe corticosteroid-induced ocular hypertension (OHT) after prolonged systemic corticosteroid use in a young woman with Takayasu's arteritis. As she did not sufficiently respond to ocular antihypertensive therapies, bilateral enhanced trabeculectomies were required to normalize her intraocular pressures. The systemic side effects of corticosteroids are well known, yet steroid-induced OHT and glaucoma remain silent causes of ocular morbidity. This case highlights the importance of IOP-monitoring in visually asymptomatic patients on systemic corticosteroids. It further emphasizes the need to raise awareness of the potential ocular side effects of steroids amongst physicians, in particular those looking after patients with autoimmune and inflammatory diseases.

## 1. Introduction

Corticosteroid-induced ocular hypertension (OHT) and glaucoma are underreported, sight-threatening complications of corticosteroid use. Patients may be asymptomatic despite very high intraocular pressures (IOPs) and advanced visual field loss.

We discuss the presentation of corticosteroid-induced OHT in a patient with Takayasu's arteritis to highlight the issues of steroid responsiveness and IOP screening in patients requiring long-term systemic steroids.

## 2. Case Report

A 22-year-old Indian woman with a past medical history of juvenile idiopathic arthritis (quiescent for seven years) and hypothyroidism presented to the medical take with a 10-day history of chest pain, palpitations, and shortness of breath. On examination, she was hypertensive at 180/100 mmHg and in pulmonary edema and had an absent left radial pulse. An echocardiogram revealed an ejection fraction of 20%. Given these findings and in the context of her autoimmune history, a diagnosis of Takayasu's arteritis was suspected. Magnetic resonance angiography (MRA) showed totally occluded left subclavian and right renal arteries with significant left renal artery stenosis, further supporting the diagnosis.

She was commenced on 80 mg oral prednisolone daily and one cycle of cyclophosphamide during a one month inpatient stay. She underwent three further cycles of cyclophosphamide as an outpatient alongside a tapering prednisolone regime of 80 mg daily, reduced to 60 mg one month later and then by a further 10 mg every month, under close outpatient monitoring.

Five months after discharge, at a rheumatology clinic follow-up, she reported a great improvement in her symptoms but complained of one episode of seeing halos. The optometrist measured an IOP of 60 mmHg in both eyes using pneumotonometry. At this point she was on 10 mg of oral prednisolone daily. She was promptly referred to eye casualty for an ophthalmology specialist opinion that confirmed abnormally high IOPs of above 50 mmHg on applanation tonometry. Despite these high IOPs, the patient was visually asymptomatic at this point in time. External examination was unremarkable. Slit lamp biomicroscopy showed deep quiescent anterior chambers, open iridocorneal angles, and healthy-looking optic discs. Given her history and examination, a diagnosis of severe corticosteroid-induced ocular hypertension was made.

Despite maximum topical therapy (prostaglandin analogue, beta-blocker, alpha-2 agonist, and carbonic anhydrase inhibitor) and selective laser trabeculoplasty to the right eye, her IOPs remained high in both eyes and were only partially responsive to oral acetazolamide. This was not a long-term treatment option owing to its side effect profile, especially given her renal disease. With her IOPs being consistently above 30 mmHg, she was at risk of developing glaucoma as well as a retinal vein occlusion. Surgical intervention became inevitable when her IOPs remained uncontrolled despite 2 g of daily oral acetazolamide and intravenous infusion of 200 mL of 20% mannitol, necessitating immediate anterior chamber paracentesis with a 30-gauge needle on two occasions. Three months following her initial presentation to eye casualty, the patient underwent bilateral mitomycin C-augmented trabeculectomies, first on the right and two weeks later on the left, to achieve satisfactory IOP control.

**Figure 1:**
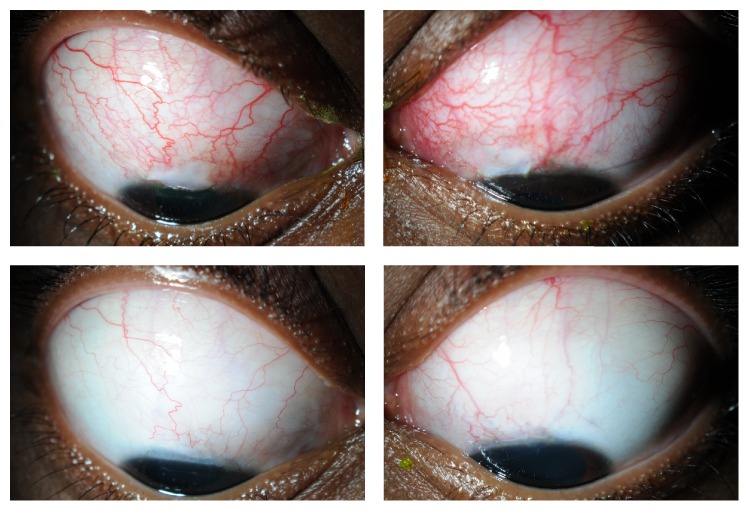
Anterior segment photographs showing the evolution of trabeculectomy blebs: one month (top panels) and four months post-op (bottom panels).

Six months postoperatively and on a tapering dose of twice daily dexamethasone 0.1% preservative-free eye drops, she has well-draining, shallow diffuse blebs with IOPs of 15 mmHg and 14 mmHg in the right and left eye, respectively (Figure 1). She no longer requires any IOP-lowering treatment. Her visual acuities, Humphrey visual fields, and optic nerve appearances remain normal. Her Takayasu's arteritis is quiescent on 7 mg of oral prednisolone daily. Since starting systemic corticosteroid therapy 12 months ago, the patient has gained 19 kg in weight.

## 3. Discussion

OHT is defined as an IOP of greater than 21 mmHg with a healthy optic disc and full visual field. After five years, 9.5% of untreated patients with OHT will develop glaucomatous optic neuropathy and associated visual field loss versus 4.4% in those receiving IOP-lowering medication [1]. Patients are usually asymptomatic but may experience episodes of blurred vision, mild (peri)ocular ache, or rings and halos when looking at bright lights.

OHT and secondary glaucoma in the context of exogenous administration of adrenocorticotrophic hormone (ACTH) for the treatment of uveitis were first described in 1950 [2]. Although the exact pathophysiology is not fully understood, glucocorticoids appear to act directly on trabecular meshwork (TM) cells to influence cellular, biochemical, and molecular changes that ultimately lead to reduced outflow facility [3, 4]. Glucocorticoids alter the organization of the actin cytoskeleton and induce the formation of so-called cross-linked actin networks (CLANs) in TM, leading to increased outflow resistance [5]. The ultrastructural changes further include an increase in myofibroblasts along the outer wall of Schlemm's canal (SC). This is compounded by increased matrix metalloproteinase (MMP) activity and an accumulation of extracellular matrix (ECM) in the juxtacanalicular connective tissue underlying the inner wall of SC [6]. ECM deposition and turnover in the TM seem to be regulated by integrin-mediated control of fibronectin matrices, and steroids have been shown to upregulate the expression of certain integrin subunits [7]. Furthermore, it has been suggested that changes in integrin expression and activity alter phagocytic function of TM cells observed in steroid-induced glaucoma [8]. In addition, and perhaps most importantly, after binding to intracellular receptors, glucocorticoids initiate signaling cascades that probably affect expression of hundreds of genes [9]. In fact, several genes have already been shown to be upregulated in dexamethasone-treated TM cells [10]. The recent identification of two independent intergenic quantitative trait loci (QTL) that could affect expression of the HCG22 mucin gene product found in trabecular meshwork cells may also soon provide further answers [11].

Corticosteroid-induced OHT and glaucoma can occur after systemic, ocular, or inhaled steroid use [12]. To date, investigators have generated corticosteroid-induced OHT and glaucoma in at least eight different species besides man, ranging from mice to nonhuman primates [3, 6]. As far as humans are concerned, sensitivity to steroids, so-called ocular steroid responsiveness, occurs in a third of the normal population [13, 14]. When treated for four to six weeks with topical corticosteroids, 5% of the normal population demonstrate a rise in IOP greater than 15 mmHg, and 30% have a rise of 6–15 mmHg, whilst the remainder show a less than 6 mmHg rise in IOP [15].

Groups of patients found to have higher rates of steroid responsiveness include those with a personal or family history of primary-open angle glaucoma, high myopia, type-1 diabetes mellitus, and connective tissue disease (particularly rheumatoid arthritis) [16]. Distribution is bimodal, with young children and older adults being most affected [16, 17]. An IOP elevation of ≥10 mmHg within three weeks has been observed in 30% of children on topical fluorometholone after strabismus surgery [18]. Given that fluorometholone is known to produce significantly fewer ocular hypertensive effects than topical dexamethasone, this is alarming, and the use of nonsteroidal anti-inflammatory drops instead of steroids after strabismus surgery in children has hence been advocated [18, 19]. Furthermore, two recent studies from India have highlighted the enormous visual disability caused by inadvertent steroid use in children, in particular, dexamethasone drops for vernal keratoconjunctivitis [17, 20].

Topical ophthalmic corticosteroids usually lead to a rise in IOP after three to six weeks of continuous administration, although a rise may be observed within one week of initiating treatment [17, 18]. In contrast, systemic steroids typically need to be administered over many months to provoke a rise in IOP [21]. The ocular hypertensive response is somewhat dose dependent and usually normalizes within a month of cessation of corticosteroid use but may be irreversible if treatment is prolonged [22]. IOP regulation in the context of intravitreal steroid injection and implantation has proven to be particularly challenging; following fluocinolone implant, up to 45% of patients eventually require glaucoma filtration surgery [23, 24].

Once steroid-induced OHT or glaucoma is diagnosed, the inciting drug should be stopped or the dosage reduced where possible. Alternatively, a different steroid formulation may be prescribed. Where steroid-induced OHT or glaucoma is irreversible, the stepwise management approach parallels that of primary-open angle glaucoma. Medical antiglaucomatous therapy comprises topical beta-blockers, alpha-agonists, carbonic anhydrase inhibitors (both topical and systemic), and prostaglandin analogues. The latter, however, may not be useful in cases of concomitant uveitic glaucoma or cystoid macular oedema. Selective laser trabeculoplasty (SLT) may potentially work as a temporizing measure in patients with steroid-induced OHT [25]. Whilst trabeculectomy and tube shunts are the preferred surgical option in adults with corticosteroid-induced OHT or glaucoma, goniotomy should be considered for initial surgical treatment in children with persistent steroid-induced glaucoma [26].

The United Kingdom National Institute of Clinical Excellence (NICE) in its 2010* “Clinical Knowledge Summary on Corticosteroids”* notes glaucoma and cataracts as adverse effects and further recommends an ophthalmic baseline assessment before initiating long-term oral corticosteroids. However, NICE fails to mention the continued risk of steroid-induced OHT and glaucoma and the associated need for repeat ophthalmic monitoring. Although both steroid-induced OHT and glaucoma are iatrogenic conditions, at least the former may not be preventable where cessation of corticosteroid therapy is impossible. However, in such cases, the treating physician should ensure an ocular steroid response is recognized before irreversible damage to the optic nerve in the form of glaucoma becomes established. Patients on topical corticosteroids usually have regular ophthalmic follow-up, and a rise in IOP is therefore picked up early. The problem arises when topical steroids are accidentally given as repeat prescriptions by the primary care physician (PCP), for example, after cataract surgery or uveitis. Good communication between the ophthalmologist and the PCP or other relevant healthcare professionals is therefore crucial. Patients requiring long-term systemic corticosteroid therapy of 10 mg or more of prednisolone daily should have their IOPs checked by an optometrist at one, three, and six months and six-monthly thereafter [22]. In addition, clinicians looking after patients on long-term corticosteroids should highlight the typically insidious nature of progressive steroid-induced OHT and glaucoma and ensure that patients understand potential warning symptoms, including halos, brow-ache, and blurred vision.
